# Making Every Contact Count with people with MSK conditions: Exploring physiotherapist acceptability

**DOI:** 10.1093/eurpub/ckac131.328

**Published:** 2022-10-25

**Authors:** A Parchment, W Lawrence, E Rahman, N Townsend, E Wainwright, D Wainwright

**Affiliations:** 1 Department for Health, University of Bath, Bath, UK; 2 Aberdeen Centre for Arthritis and MSK Health, University of Aberdeen, Aberdeen, UK; 3 MRC Lifecourse Epidemiology Unit, University of Southampton, Southampton, UK; 4 Public Health Workforce Development, Health Education England Wessex, Winchester, UK

## Abstract

There are known risk factors that are associated with the onset and exacerbation of musculoskeletal (MSK) conditions and pain. Physiotherapists are uniquely placed to deliver brief interventions with their patients. Healthy Conversation Skills is the main training component of the Wessex approach to Making Every Contact Count. Despite its potential for promoting MSK health and wellbeing, there is no evidence to support its acceptability within MSK services. This is the first known study to explore the use and perceptions of the Wessex model of MECC HCS within MSK services. A mixed method design was used. Phase one employed an online questionnaire, open to all professionals trained in MECC HCS, consisting of items relating to implementation outcomes. Barriers and facilitators to delivery were explored and mapped to the Theoretical Domains Framework. Phase two invited physiotherapists for a follow-up interview and qualitatively explored their acceptability of delivering MECC HCS to patients with MSK conditions. MECC HCS was found to be highly acceptable, appropriate, and feasible. Physiotherapists reported using their skills at least daily but missed opportunities for delivering MECC HCS were evident. Barriers mapped mostly to ‘Environmental Context and Resources’ on the Theoretical Domains Framework. Qualitative themes developed during phase two were: ‘Recognising the patient as the expert supports change', ‘MECC HCS improves physiotherapy practice', ‘MECC HCS shared problem solving reduces workload', ‘time as a perceived barrier to MECC HCS’ and ‘system-level support needed to sustain MECC HCS'. MECC HCS is a promising brief intervention for supporting people with MSK conditions. Further rollout of this intervention may be beneficial for meeting the goals of the NHS and Public Health England in prevention of MSK conditions and promotion of MSK health. Barriers associated with sustainability must, however, be addressed.

**Key messages:**

• Making Every Contact Count Healthy Conversation Skills is considered a highly acceptable brief intervention for supporting behaviour change in people with musculoskeletal conditions.

• Organisational, system-level barriers to implementation must be addressed in order to increase sustainability and enhance future roll out of the brief intervention.

